# Spectrum of early lung cancer presentation in low-dose screening CT: a pictorial review

**DOI:** 10.1007/s13244-016-0487-4

**Published:** 2016-05-17

**Authors:** Cristiano Rampinelli, Sonia Francesca Calloni, Marta Minotti, Massimo Bellomi

**Affiliations:** Department of Medical Imaging and Radiation Sciences, European Institute of Oncology, Via Ripamonti, 435, 20141 Milan, Italy; School of Medicine, University of Milan, Milan, Italy

**Keywords:** Lung cancer, Low-dose CT, Screening, Pulmonary nodule

## Abstract

The typical presentation of early stage lung cancers on low-dose CT screening are non-calcified pulmonary nodules. However, there is a wide spectrum of unusual focal abnormalities that can be early presentations of lung cancer. These abnormalities include, for example, cancers associated with ‘cystic airspaces’ or scar-like cancers. The detection of lung cancer with low-dose CT can be affected by the absence of intravenous contrast medium. As a consequence, endobronchial and central lesions can be difficult to recognize, raising the potential for missed cancers. Focal lesions arising within pre-existing lung disease, such as lung fibrosis or apical scars, can also be early lung cancer manifestations and deserve particular consideration as recognition of these lesions may be hindered by the underlying disease. Furthermore, the unpredictable growth rate of lung cancer, which ranges from indolent to aggressive cancers, necessitates attention to the wide spectrum of progression in lung cancer appearance on serial low-dose CT scans. In this pictorial review we discuss the spectrum of early lung cancer presentation in low-dose CT screening, highlighting typical as well as unusual radiological features and the varied growth rates of early lung cancer.

*Teaching Points*

• *There is a wide spectrum of early presentations of lung cancer on LDCT.*

• *Low radiation dose and the absence of contrast medium injection can affect lung cancer detection.*

• *Lung cancer growth shows various behaviours, ranging from indolent to aggressive cancers.*

• *Familiarity with LDCT technique can improve CT screening effectiveness and avoid missed diagnosis.*

## Introduction

Lung cancer is the most commonly diagnosed cancer, and was the leading cause of cancer death globally in males in 2008; among females, it was the fourth most commonly diagnosed cancer and the second leading cause of cancer death [1].

The National Lung Cancer Screening Trial (NLST) has recently demonstrated that low-dose computed tomography (LDCT) reduces lung cancer mortality by 20 % in high-risk subjects compared to chest X-ray screening [2]. Based on this result, LDCT screening of heavy smokers has recently been recommended by the major American and European scientific societies [3–6].

LDCT of the lung would seem to be a simple radiological interpretation but the absence of contrast material injection and low signal-to-noise ratio due to the low radiation exposure can lead to misinterpretation and missed significant findings.

Moreover, the distinction between different types of pulmonary nodules, awareness of the behaviours of early stage lung cancer and the interpretation of serial LDCT scans are fundamental in the management of the findings.

In this pictorial review, we discuss the wide spectrum of early LDCT findings in lung cancer screening, highlighting typical as well as unusual radiological features and the varied growth rates of early stage lung cancer.

### Low-dose CT protocol

LDCT for screening purposes is an unenhanced, single breath-hold scan, conducted from the lung apices through the lung bases.

Low-dose protocols of the chest can be achievable due to a high contrast resolution between the air and lung nodules, gaining a low radiation dose while maintaining good diagnostic quality.

There is no consensus on which level of dose is considered a ‘low-dose’ CT, however, the major screening trials used tube voltages between 90 kVp and 140 kVp and tube current in the range of 30–80 mAs [7]. Both parameters should be combined to obtain an acceptable radiation dose, taking into account the patient’s body habitus and age. The American College of Radiology (ACR), suggest a CTDIvol ≤3 mGy for standard size patients [8] while an NLST study concluded that acceptable CT screening can be accomplished at an overall average effective dose of approximately 2 mSv [9]. The National Comprehensive Cancer Care Network guidelines have suggested an effective dose of ≤3 mSv for patients with a body mass index (BMI) ≤30 kg/m2 and ≤5 mSv for patients with a BMI >30 kg/m2 [10].

A gantry rotation time of <0.5 seconds is recommended by the ACR [8]: faster rotation is associated with fewer motion artifacts and a decreased radiation dose to the patient.

LDCT collimation should be set with the purpose of achieving thin-section reconstruction images, of at least ≤1.5 mm [8]. Thin-section images are essential in reading and interpretation of low-dose CT scans, allowing the use of a CAD system, volumetric analysis and a more accurate evaluation of subsolid nodules.

Most LDCT screening studies have used the filtered back projection technique for image reconstruction; nevertheless, iterative reconstruction algorithms have been shown to improve image quality while reducing radiation dose [11–13]. Recent studies have shown that iterative reconstruction algorithms allow the use of low-dose CT and ultra-low-dose CT with acceptable image quality [14, 15] and that the detection of subsolid nodules is not affected by such algorithms [14, 16].

Various methods and tools are available in reporting chest low-dose CT. Beside the evaluation of axial thin-section images, the detection rate of pulmonary nodules can be improved using maximum intensity projection (MIP) which consists of projecting the voxel with the highest attenuation value on every view throughout the volume onto a 2D image. Bastarrika et al. [17] showed that non-overlapping, 10-mm-thick axial MIP reconstructions enabled more accurate detection of pulmonary nodules in comparison with 1.25-mm conventional axial images. Furthermore, Jankowski et al. evaluated the diagnostic benefits of a MIP and CAD system for pulmonary nodule detection compared with 1-mm LDCT images and found that MIP and CAD reduced the number of overlooked nodules [18]. Also, multiplanar reconstruction images (MPR) were shown to be a sensitive technique in lung nodule detection [19], which can be particularly useful for evaluation of lung hilum and apex.

The use of CAD systems can improve radiologists detection rate for small pulmonary nodules [20]. The accuracy of CAD for nodule detection increases with thinner reconstruction images and with overlapping reconstructed sections [21], even if this causes an increasing number of false-positive results.

However, to date, there is no consensus on the optimal reading technique; further investigations are needed to assess which method should be used to improve the detection rate of pulmonary nodules on low-dose CT.

### Lung cancer presenting as lung nodules

Low-dose CT can identify small pulmonary nodules in heavy smokers and thus improve the detection of lung cancer at an earlier and potentially more curable stage [22, 23]. Non-calcified pulmonary nodules are the typical presentation of screen-detected lung cancers on low-dose CT. Based on the definition of the Fleischner Society [24], the pulmonary nodule is a rounded or irregular opacity, well or poorly defined, measuring up to 3 cm in diameter.

According to their attenuation, lung nodules can be classified as solid or subsolid, the latter further divided into part-solid or non-solid nodules [25].

**Fig. 1:**
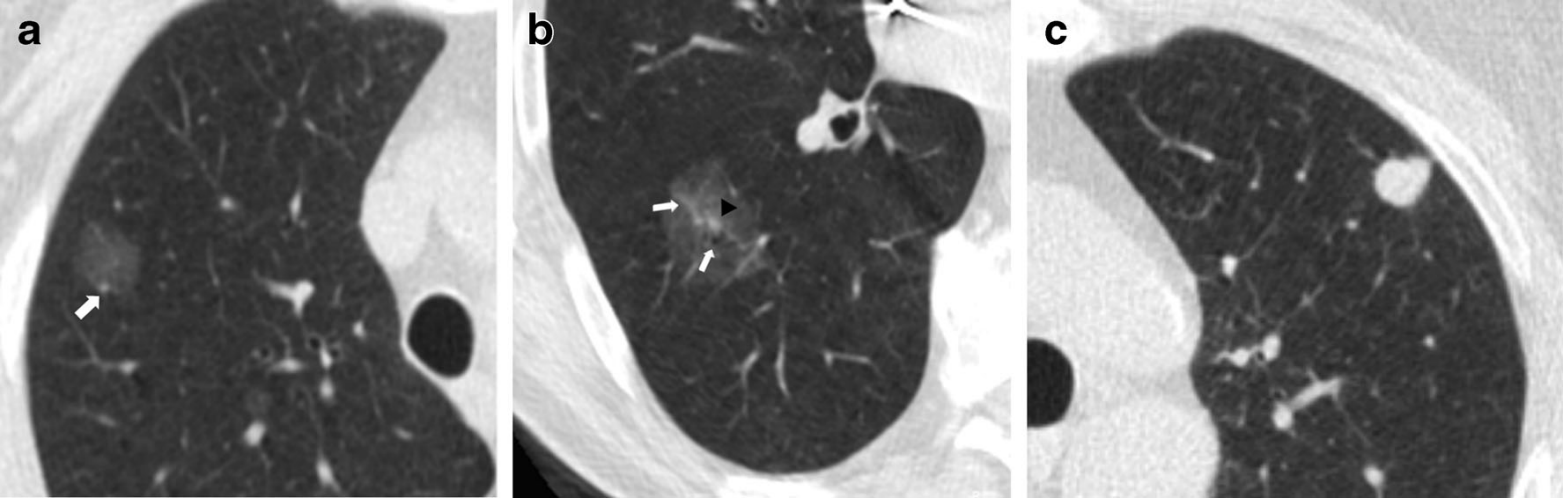
Primary lung adenocarcinomas. Radiological features of pulmonary nodules according to their attenuation. (**a**) A low-dose CT thin-section shows a 15-mm non-solid nodule (pure ground-glass attenuation) of the right upper lobe: the nodule density is slightly higher than that of normal lung parenchyma, and vessels are visualized within the nodule (*arrows*). (**b**) Low-dose CT scan shows a 25-mm part-solid nodule of the right lower lobe. This nodule is characterized by a 3-mm central solid component (*arrowhead*) surrounded by a pure ground-glass opacity. The vessels and a small peripheral airway (*arrow*) can be clearly depicted within the ground-glass opacity. (**c**) Axial low-dose CT scan shows a 12-mm subpleural solid nodule of the left upper lobe. The spectrum of early stage lung cancer presentation at low-dose CT includes solid nodules as well as part-solid nodules and ground-glass nodules

The spectrum of early stage lung cancer presentation at low-dose CT includes solid nodules as well as part-solid nodules and ground-glass nodules (Fig. 1).

A pulmonary nodule is defined as solid when it completely obscures the entire lung parenchyma within it. Non-solid nodules, or pure ground-glass nodules (pGGN), are defined as focal nodular areas of increased attenuation through which lung parenchymal structures, such as the pulmonary vessels or airway structures, can be visualized. Part-solid nodules are those having both ground-glass and solid components.

Lung nodule attenuation is, therefore, a key feature in lung cancer screening, with particular emphasis on subsolid nodules. Subsolid nodules are complex lesions both for interpretation and management, and recently the Fleischner society has published recommendations for their management [26]. Of subsolid nodules, 40–70 % resolve spontaneously in 3 months and, upon resolution, are presumed to have been benign [27]. Part-solid nodules that persist at 3-month follow-up should be considered potentially malignant: in the ELCAP study, after adjusting for nodule size, the malignancy rates for part-solid, solid and pGGN were 63 %, 32 % and 13 %, respectively [25].

**Fig. 2:**
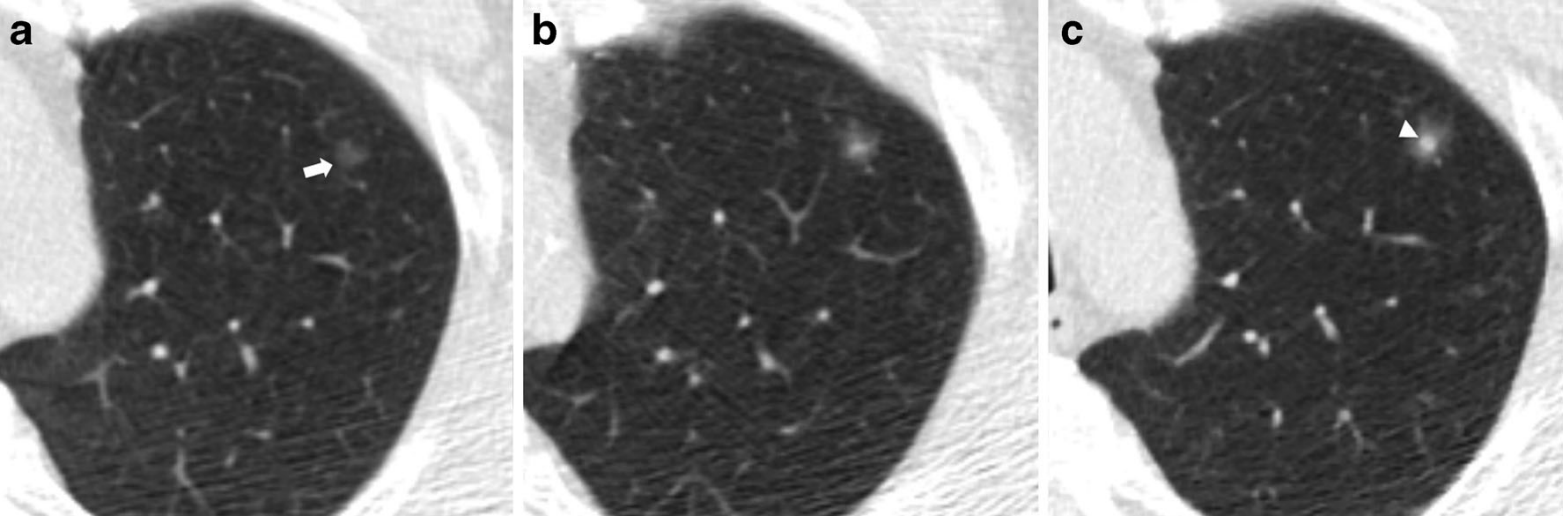
Increased attenuation of a small lung adenocarcinoma over 3 years in a 62-year-old man. (**a**) Low-dose CT image show a 5-mm non-solid nodule of the upper left lobe (arrow). (**b**) Follow-up CT obtained 1 year later show a minimum increment of axial diameter and a slight increase of nodule attenuation. (**c**) Two years later the progression to a part-solid nodule is obvious. The development of a central solid component (*arrowhead*) within the nodule can be observed. An increase in pulmonary nodule attenuation at LCDT follow-up, the development or increment of a solid component within the pulmonary nodule should raise suspicions of malignancy, even if the nodule size is stable. Typically, this appearance is suggestive of a peripheral lung adenocarcinoma

**Fig. 3:**
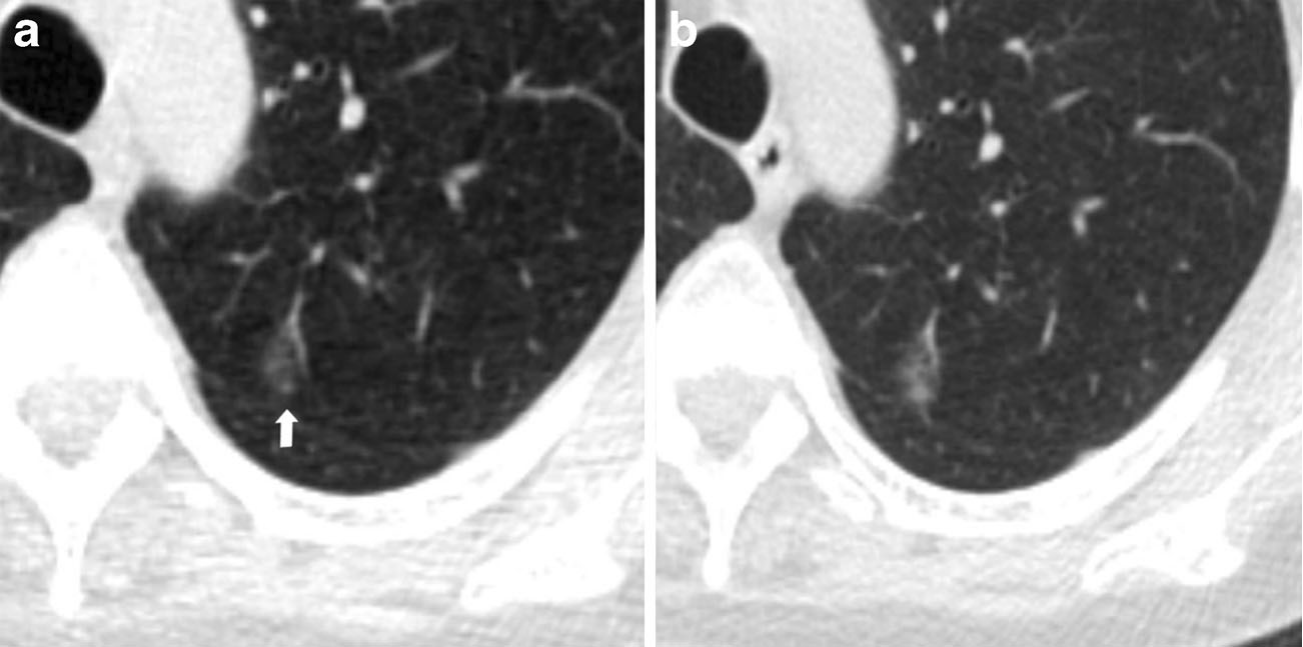
Progression at the 1-year interval of a non-solid nodule in a 66-year-old woman. (**a**) Low-dose CT section shows a 12-mm nodule with ground-glass attenuation of the upper left lobe (arrow). (**b**) CT scan obtained at annual repeat LDCT demonstrates a substantial stability in size but with increased density, that indicates an increased risk for malignancy. The lesion was surgically removed with a diagnosis of lung adenocarcinoma. Sub-solid nodules that show an increment in attenuation should be considered suspicious for malignancy

An increase in pulmonary nodule attenuation at LCDT follow-up, the development or increment of a solid component within the pulmonary nodule should raise suspicions of malignancy, even if the nodule size is stable (Figs. 2 and 3).

The pulmonary nodule size is another important predictor for malignancy in heavy smokers, as good evidence exists for a strong correlation between nodule size and risk of malignancy [28, 29]: in a meta-analysis of eight CT screening trials, the prevalence of malignancy depended on nodule size, ranging from 0 % to 1 % for nodules <5 mm, from 6 % to 28 % for those between 5 and 10 mm, and from 64 % to 82 % for nodules > 20 mm [30]. A recent study by Horeweg et al. [31] reported the probability of developing lung cancer in participants from the Dutch–Belgian Lung Cancer Screening (NELSON) trial. The authors found that participants with a nodule smaller than 5 mm had a very low risk (0.6 %) of developing lung cancer during 2 years of follow-up.

### Unusual presentations of lung cancer

Besides the typical presentation of lung cancer, e.g. pulmonary nodule, there is a wide spectrum of focal abnormalities seen on low-dose CT that could not truly be defined as a pulmonary nodules but, nevertheless, are early presentations of primary lung cancer. These kinds of abnormalities, which are unusual forms compared to pulmonary nodules, include cancers associated with ‘cystic airspaces’ or ‘non-nodular’ cancers presenting, for example, as fibrotic changes or scar-like lesions. Such focal abnormalities are initial signs of lung cancer which usually develop over time in a lung nodule or suspicious pulmonary opacities and, therefore, should be checked at follow-up or annual repeat LDCT.

There is increasing evidence that early stage lung cancer could arise from pre-existing pulmonary ‘cystic airspaces’ [32–35], including in this description all findings associated with air spaces, such as cysts, bullae, and blebs.

These cancers associated with cystic airspaces have recently been described in a lung cancer screening program [36]: out of 26 lung cancers identified abutting or in the wall of a cystic airspace, 13 were identified at baseline (13/595, 2 %) and 13 at annual screening (13/111, 12 %), which was significant (*p* < 0.0001). In a recent analysis aimed at evaluating findings of 22 interval and post-screen carcinomas in lung cancer screening, the authors showed that 22 % (5/22) of missed cancers presented as bulla wall thickening on LDCT [37, 38].

**Fig. 4:**
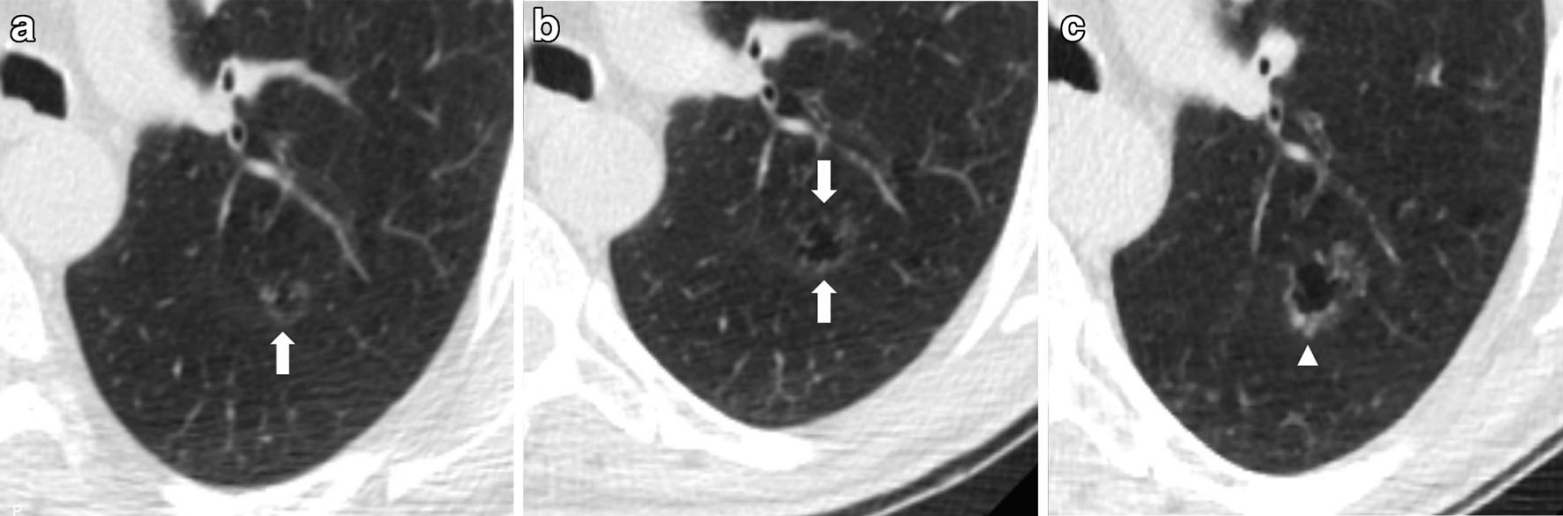
Progressive changes of a lung adenocarcinoma associated with a cystic airspace in a 60-year-old woman. (**a**) Axial low-dose CT shows an 8-mm cystic airspace with thin irregular non-solid wall in the upper left lobe (*arrow*). (**b**) One year later the lesion has increased in size to 12 mm and 2 years later, (**c**) a consistent irregular thickening with the development of solid components along the wall was evident (*arrowhead*). The histologic diagnosis was lung adenocarcinoma. This kind of presentation is usually slow growing on serial low-dose CT scans and the diagnosis of lung cancer is suggested by the circumferential wall thickening of the cystic airspaces; with tumor growth, airspaces size could increase, decrease or remain unchanged

This kind of presentation is usually slow growing on serial low-dose CT scans [26] and the diagnosis of lung cancer is suggested by the circumferential wall thickening of the cystic airspaces or by the arising of a nodule along the wall of the cystic airspaces (Fig. 4). Nevertheless, with tumor growth, airspaces size could increase, decrease or remain unchanged [35].

**Fig. 5:**
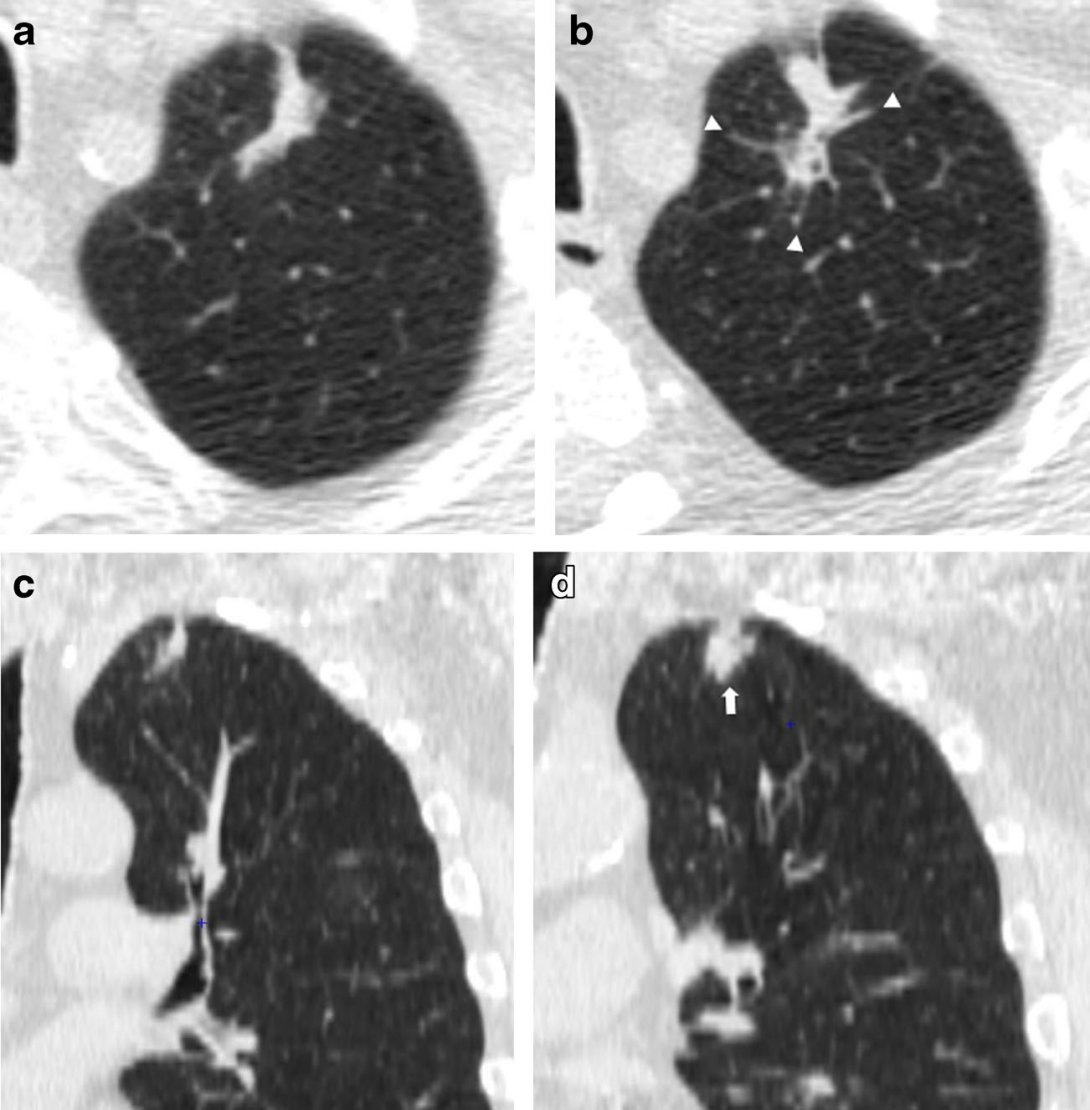
Low-dose CT images show both an increase in size and density of a scar-like lesion of the left lung apex, consisting of a lung adenocarcinoma. (**a**, **c**) Axial and coronal LDCT sections of the left apex show an irregular subpleural opacity that was interpreted as apical fibrotic changes. (**b**, **d**) LDCT repeated at a 1-year interval shows spiculations (*arrowhead*) and a significant thickening of the lesion (*arrow*). A PET/CT scan was positive and the patient went to lobectomy with histologic diagnosis of adenocarcinoma. Lung cancers presenting as fibrotic changes or as scar-like lesions are relatively uncommon but could be encountered in LDCT screening

Lung cancer presenting as fibrotic changes or as scar-like lesions are less common but could be encountered in LDCT screening (Fig. 5). In a recent article aimed at evaluating the early features of incident lung cancers detected with LDCT [39], a remarkable percentage (7/17, 41 %) of cancers which exhibited an initial ‘non-nodular’ shape were observed. Similarly, Xu et al. [40] reviewed the images of patients with diagnoses of lung cancer in annual repeat rounds of CT screening and showed that some cancers were recognized at previous LDCT scans but they were considered to represent benign rather than malignant findings. In that study, the cancers were misclassified as fibrosis, scarring, and a simple bulla, which the authors attributed to a lack of experience in identifying findings suspicious for small lung cancers or identifying small cancer with an unusual appearance.

### Lung cancer growth

One of the advantages in lung cancer screening is the availability of serial annual LDCT scans, permitting comparison of early changes of suspicious findings.

The growth rate of CT screen-detected lung cancer is often unpredictable, with many characteristics such as size, morphology, and attenuation that should be taken into account managing suspicious lung nodules. Early lung cancers could present various behaviours at LDCT follow-up, ranging from very slow growth to rapidly growing cancers.

Growth rate can be difficult to estimate and volumetric analyses of pulmonary nodules has been suggested as a precise tool for nodule size measurement and an accurate method for assessing nodule growth. In the NELSON trial, solid intraparenchymal nodules were measured by volumetric software and with semi-automated measurements, the interobserver agreement was excellent: identical volumes were measured by both observers in 89 % of nodules. However, in a minority (approximately 11 %) of small solid nodules, semiautomated measurements were not completely reproducible and, thus, may cause errors in the assessment of nodule growth. The most frequent cause of variability was incomplete segmentation performed by the software due to an irregular shape or irregular margins of the nodule. For this reason, for small or irregularly shaped nodules, an observer should check the segmentation shown by the program before accepting the semiautomated volume measurement and drawing conclusions about growth or stability of a nodule [41].

Obviously, the behaviours of screen-detected lung cancer is strongly related to its histology: small cells and squamous cell lung cancers are usually more aggressive forms compared to adenocarcinomas which have a wide range of growth but are usually slower [42].

**Fig. 6:**
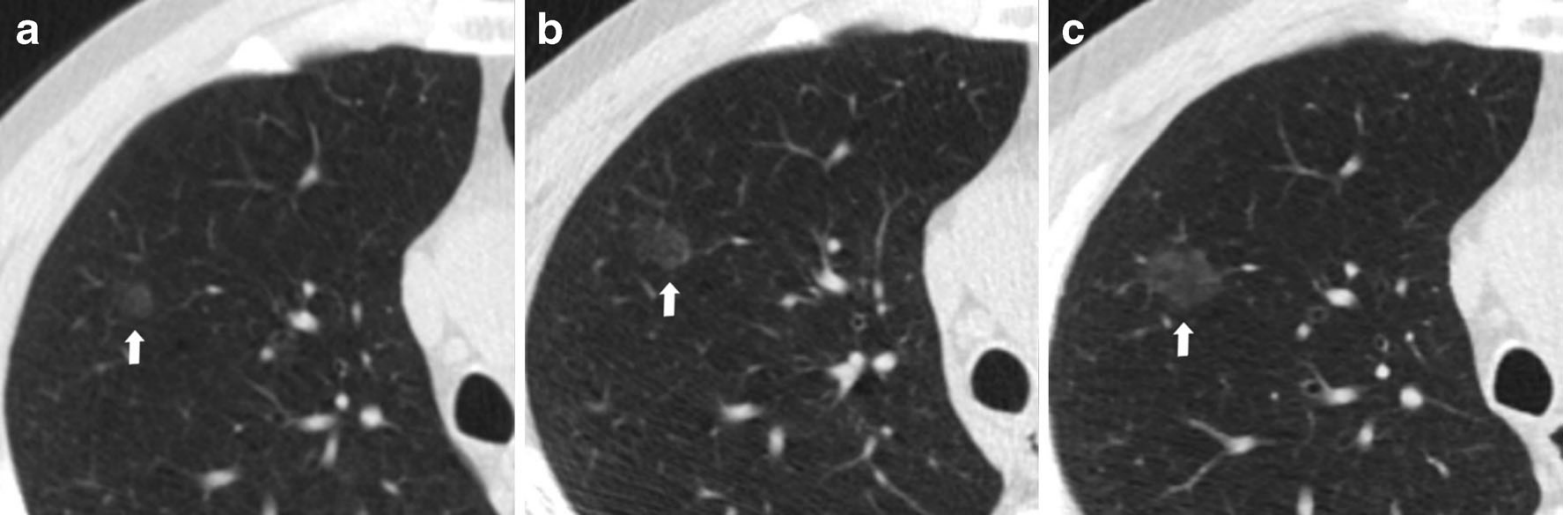
Slow-growing lung adenocarcinoma in a 61-year-old man. (**a**) Low-dose CT axial section in the right upper lobe shows a 6-mm ground-glass nodule (*arrow*). (**b**) Low-dose CT scan after 2 years shows an increase in size of the nodule to 10 mm (*arrow*). (**c**) After 4 years from the first low-dose CT scan, the nodule showed the same ground-glass attenuation but a progressive increase in size from 10 to 14 mm (*arrow*). Typically, early lung cancer presenting as non-solid nodules are slow-growing forms

Tipically, early lung cancer presenting as non-solid nodules (ground-glass nodules, GGN) are slow-growing forms. Such cancers, even if nodule size is determinant, could have such indolent behaviours that short-term LDCT follow-up (e.g. 3 months) could be insufficient to determinate the real growth, and longer follow-ups (e.g. up to 3 years) are desirable to assess nodule growth (Fig. 6) [26]. When growth is very slow and previous LDCT scans are available, it is necessary to make annual comparisons with the oldest LDCT scan available to carefully note slight changes over time.

Part-solid nodules, especially those in which the solid component is larger than 5 mm, should be considered malignant until proven otherwise, provided either growth or no change is seen at the 3-month LDCT follow-up . For these complex lesions, the growth of the solid portion is more important and reliable in assessing the malignancy of the nodule (Fig. 2).

**Fig. 7:**
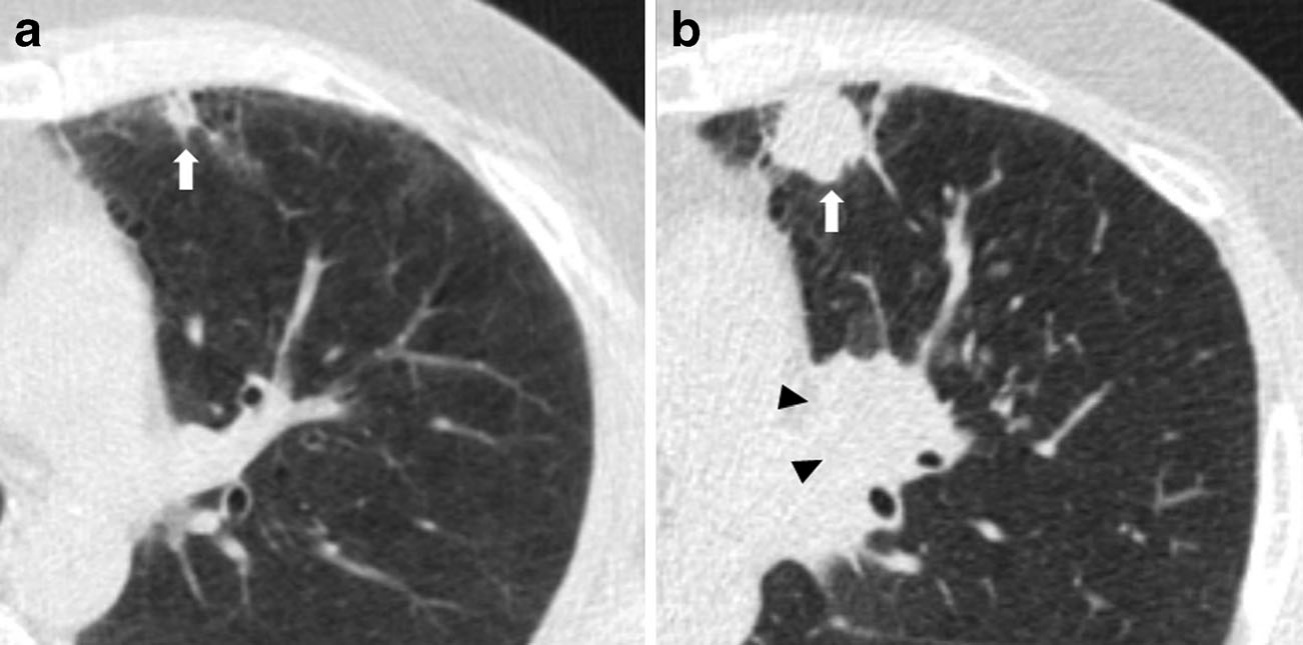
Fast-growing lung adenocarcinoma in a 79-year-old man. (**a**) Axial LDCT image of the chest shows a 7-mm sub-pleural solid nodule in the left upper lobe (*arrow*). (**b**) LDCT scan obtained at 1-year interval shows a rapid growth of the lesion into a 23-mm solid nodule (*arrow*) with an aggressive behavior documented by the concomitant enlargement of hylar lymph nodes (*arrowheads*)

Solid nodules have a wide range of growth but usually present faster growth compared to subsolid nodules [43, 44] (Fig. 7).

**Fig. 8:**
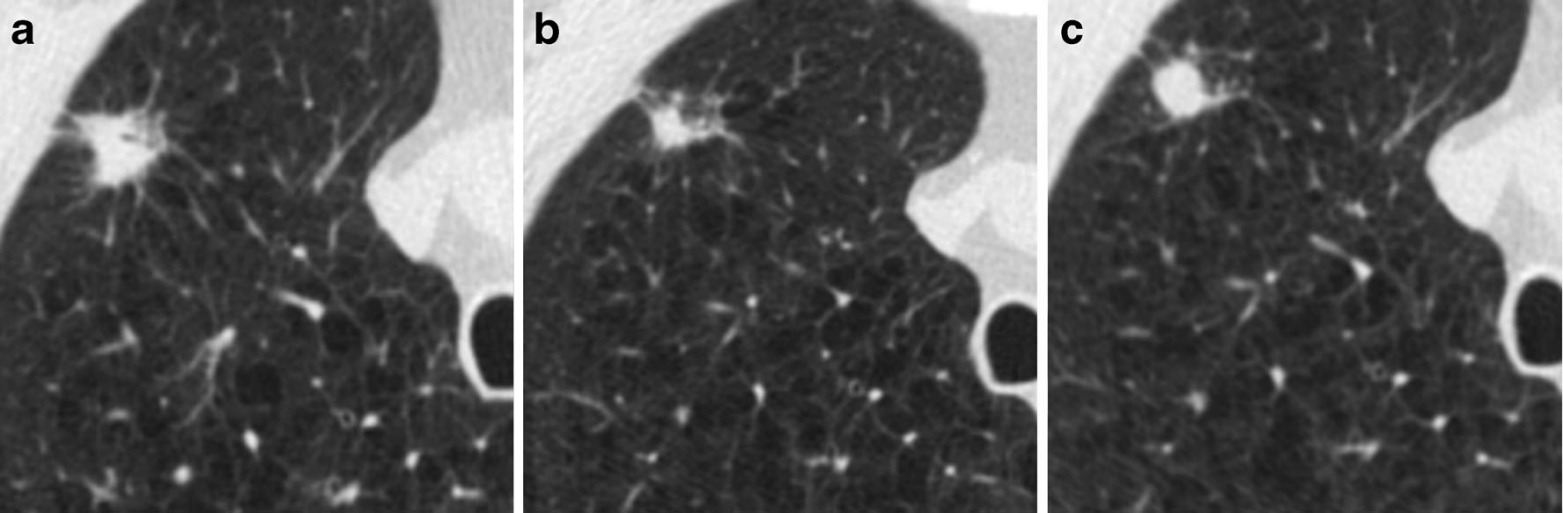
Transient decrease in size of a squamous cell lung cancer in a 74-year-old man. (**a**) Unenhanced axial LDCT image of the chest shows a solid nodule in the upper right lobe with an irregular shape and a maximum diameter of 19 mm. (**b**) Follow-up LDCT scan obtained 1 month after antibiotic therapy demonstrated a significant regression of the lesion. (**c**) At LDCT obtained 3 months later, the nodule grew in size again. Biopsy was performed, and results of histology revealed lung adenocarcinoma. It is reported that adenocarcinomas can show a transient decrease in size, maybe related to fibrosis or atelectasis. In this cases, a further LDCT scan is suggested to assess the complete resolution or stability of the findings and to avoid missed diagnosis

It is also important to consider that care should be taken not to assume that all lesions that partially decrease in size at short-term LDCT follow-up are necessarily benign. It is documented that adenocarcinomas can decrease temporarily in size after antibiotic therapy, owing to fibrosis or atelectasis [26]; in this case, a further LDCT scan is suggested to assess the complete resolution or stability of the findings and to avoid missed diagnosis (Fig. 8).

### LDCT: lesion location and cancer detection

The detection of lung cancer with low-dose CT may be hindered by the absence of intravenous contrast medium and the low signal-to-noise ratio. As a consequence, endobronchial lesions, pulmonary-hilum lung cancers, and parenchymal abnormalities at lung apices or within lung fibroses are difficult to recognize, raising the potential for missed cancers.

Even if small lung cancer detected by screening are more frequently localised in peripheral lung areas, nevertheless, a small amount of lung cancers are also centrally located or endobronchial.

The majority of screen-detected lung cancers are localised in the periphery of the lungs, represented by the large amount of adenocarcinomas more often localised peripherally [31], while squamous cell and small cell carcinomas are most common centrally. A retrospective study performed by Lindell and colleagues [45] showed that squamous cell and small cell carcinomas represented 71 % and 88 %, respectively, of central lung lesions (11 % of cancers detected).

**Fig. 9:**
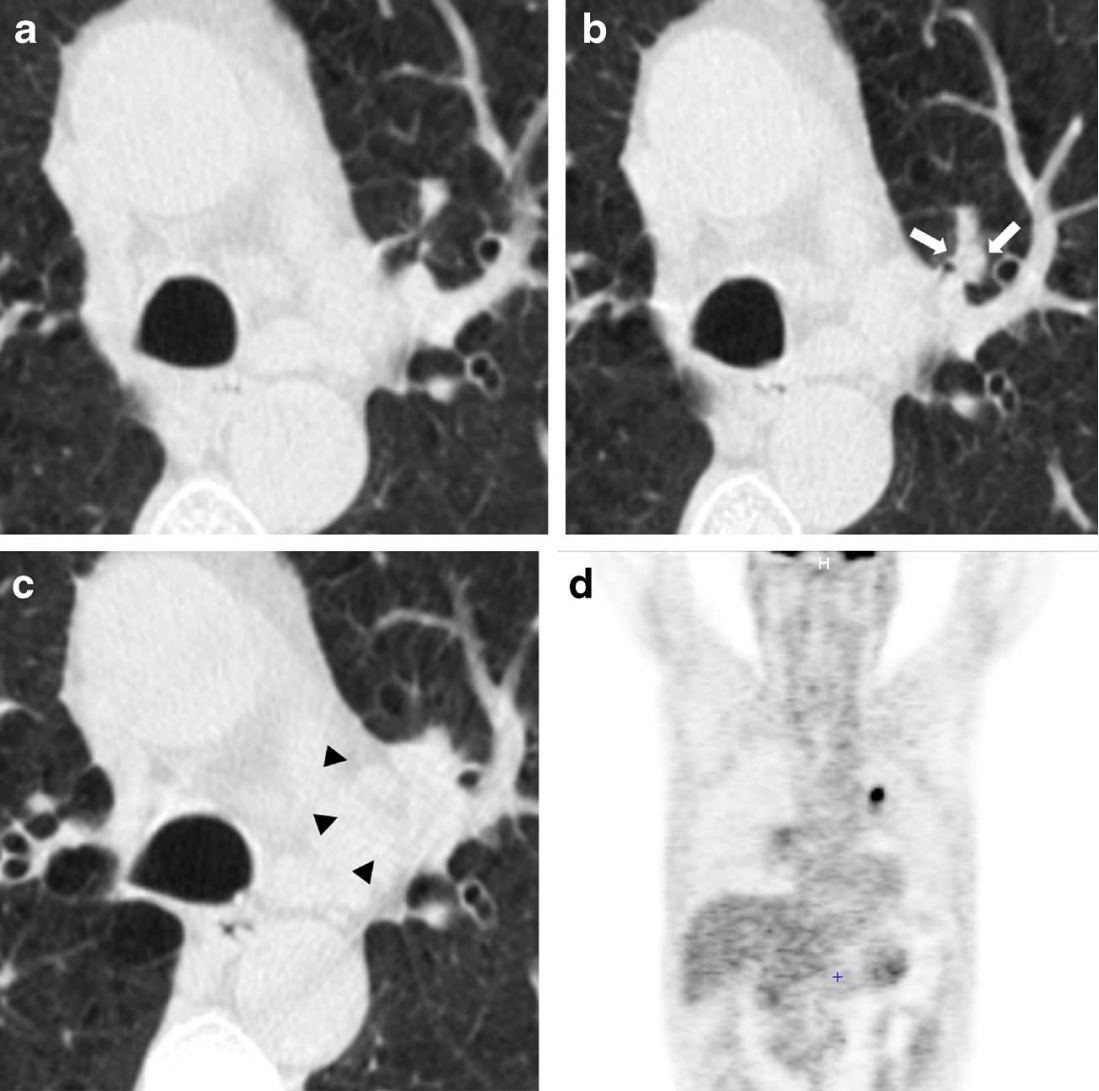
Lung cancer of the left hylum in a 67-year-old man with history of sarcoidosis. (**a**) Axial low-dose CT image through lung hyla does not show any significant findings at baseline CT screening. (**b**) Low-dose CT image obtained at the same level 1 year later shows the appearance of a 6-mm pulmonary nodule (*arrow*) surrounded by vascular and airways structures of the left hylum. This finding without contrast material injection can be misinterpreted as a pulmonary vessel. (**c**) The following year, the low-dose CT scan reveals the presence of a large lesion of the left hylum (*arrows*) that was characterize by increased metabolic activity via PET scan (**d**). The histological diagnosis was a T2N1 squamous cell carcinoma

**Fig. 10:**
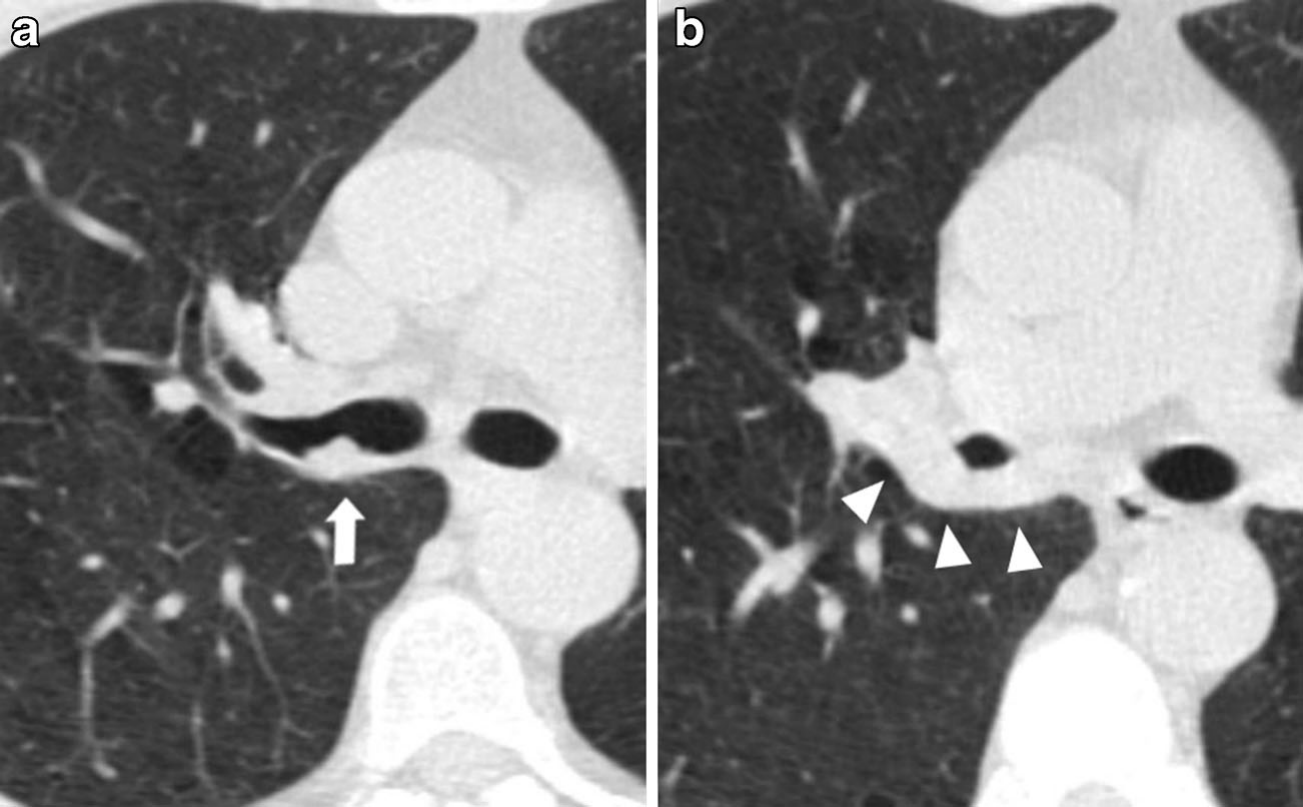
Endobronchial lung cancer. (**a**) Axial low-dose CT shows a small lesion abutting the posterior wall of the right main bronchus, interpreted as non-suspicious bronchial mucus (*arrow*). (**b**) Low-dose CT performed 1 year later shows a significant reduction of bronchial caliber due to the presence of neoplastic tissue (*arrowheads*)

Compared to peripherally located carcinomas, such lung cancers could be difficult to detect because low-dose CT images are unenhanced scans and possibly because screening a population at this location is far less common. In a recent publication by Scholten and colleagues [46] focused on missed lung cancer in the NELSON screening trial, the authors showed that more than one-third of the 22 missed cancers were endobronchial lesions (5/22) or mediastinal/hilar lymph nodes (3/22). A dedicated evaluation of endobronchial and hilar regions, with the use of coronal MPR and both lung and mediastinal window settings, is, therefore, suggested to minimize detection errors in lung cancer screening (Figs. 9 and 10). Furthermore, endobronchial and central lesions are mostly represented by fast-growing or fast-progressing tumors such as squamous cell carcinomas and small cell carcinomas [47] with a usual VDT shorter than adenocarcinoma’s [48], making the early diagnose of these lesions a worthy challenge, as these rapidly growing missed cancers have the potential to affect patient prognoses.

Additional lung regions that deserve specific consideration in lung cancer screening are the lung apices. The detection and interpretation of parenchymal abnormalities within lung apices could be a challenge in lung cancer screening, as apical regions are a frequent location of fibrotic scars and differential diagnosis between lung cancer and fibrotic changes could be complicated. Indeed, as reported by Scholten and colleagues [46], an apical scar-like nodule was identified as a cause of a missed lung cancer due to an interpretation error. Similarly, Diederich and colleagues [49] reported one nodule interpreted as an apical scar at initial LDCT that showed slow growth after 24 months was revealed to be lung adenocarcinoma.

**Fig. 11:**
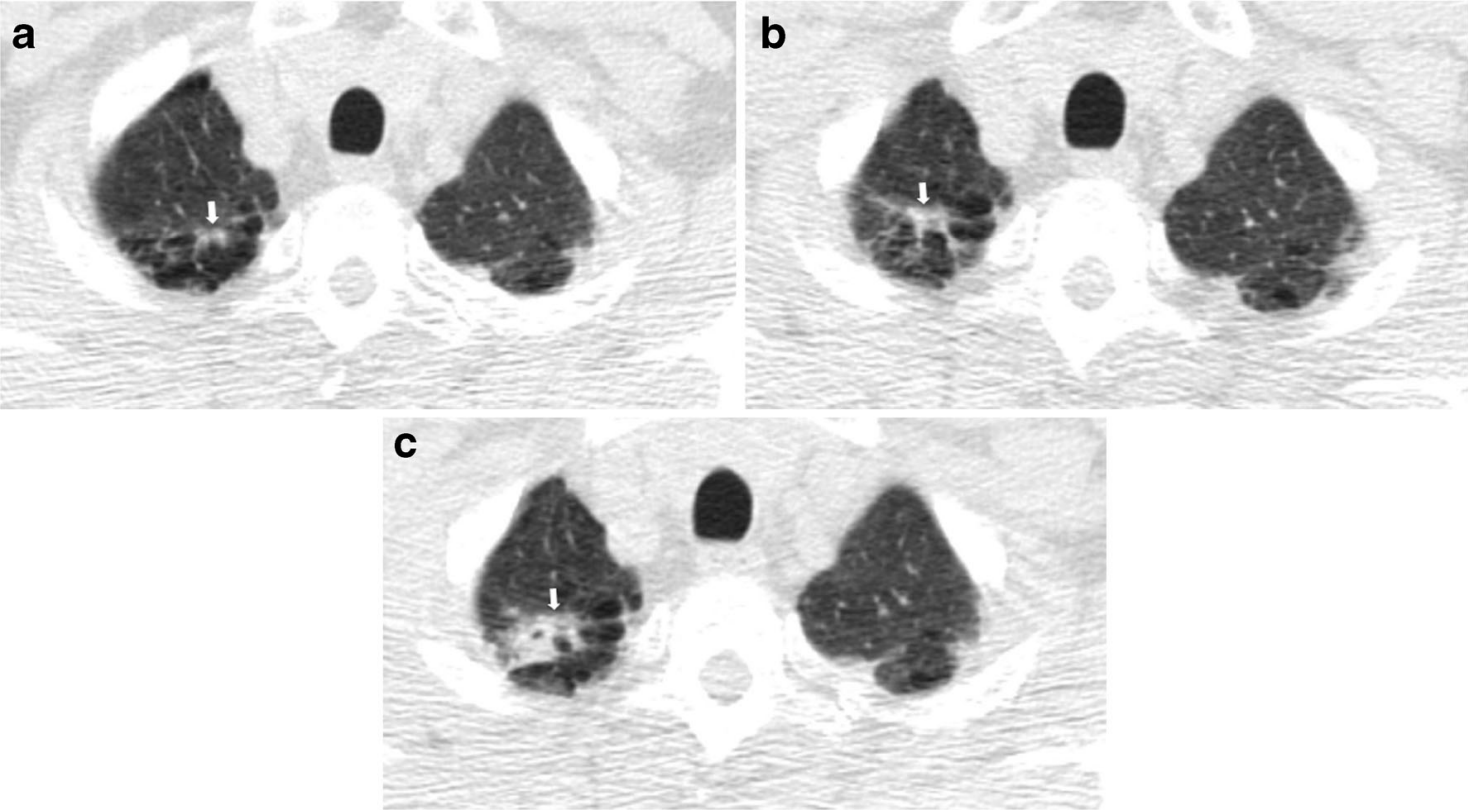
Right apical lung cancer in a 71-year-old man. (**a**) Low-dose axial CT scan shows small irregular fibrotic changes of the right apex (*arrow*). (**b**) One year later, LDCT shows a slight increment of the focal abnormalities with the typical aspect of apical fibrotic changes, with a pleural tag and concomitant emphysema (*arrow*). (**c**) Two years later, a remarkable increment in size was clearly depicted (*arrow*) and the lesion has been surgically removed with a diagnosis of lung adenocarcinoma

Lung cancer diagnosis should, therefore, be taken into account when a new lesion arises in a previously detected apical scar or when a focal apical abnormality shows progressive enlargement at annual repeat low-dose CT scans. Any change over time within an apical scarring should be carefully evaluated to detect potential lung cancer (Fig. 11).

**Fig. 12:**
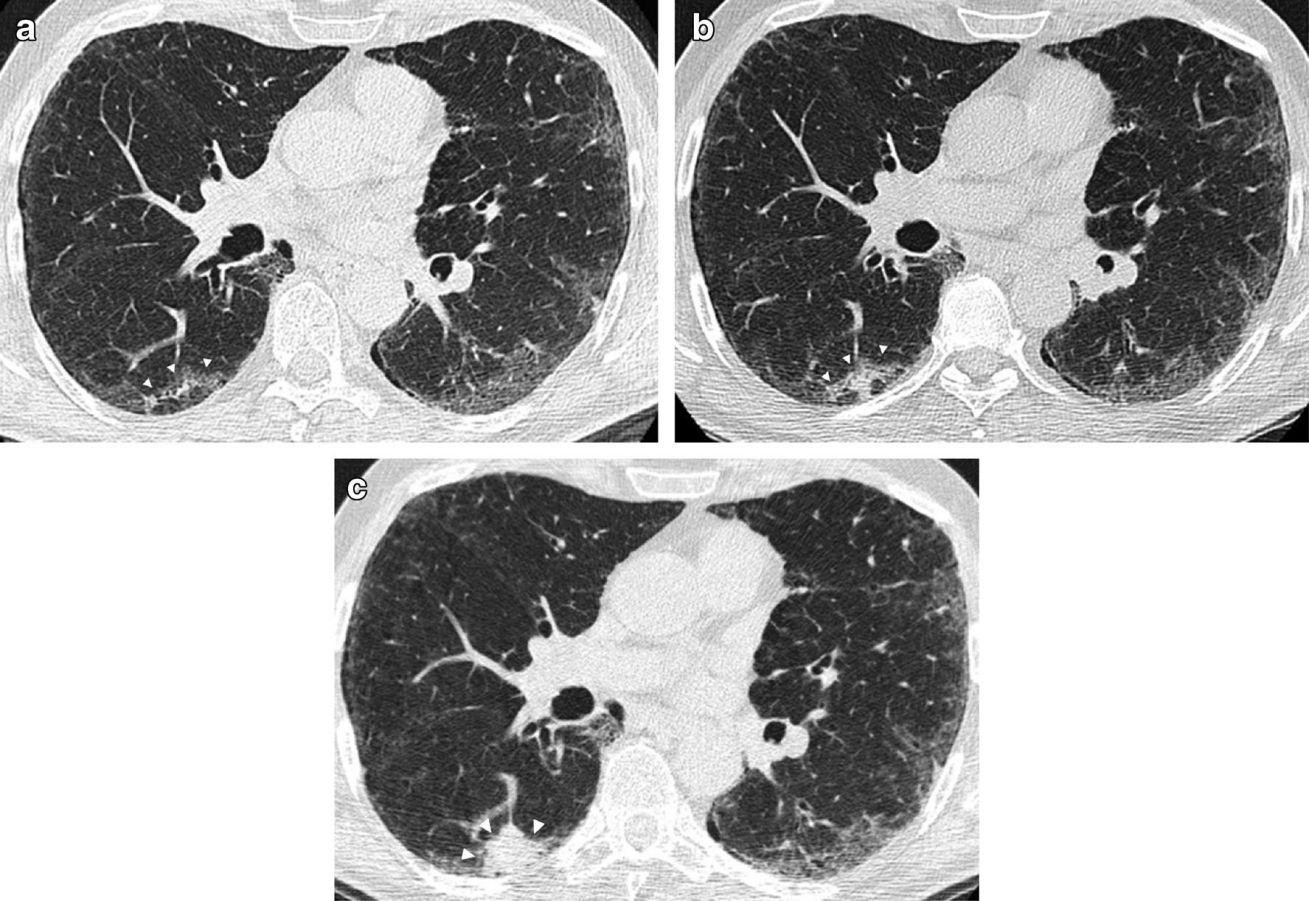
Lung cancer arising within a pre-existing fibrotic lung disease. (**a**) Low-dose CT demonstrates a bilateral subpleural reticular pattern and a ground-glass opacity, with a subpleural curvilinear opacity in the right lower lobe (*arrowheads*). (**b**) Low-dose CT scan obtained 1 year later confirmed the fibrotic disease with an increased density of the subpleural opacity in the right lower lobe (*arrowheads*), interpreted as a progression of the fibrosing pattern. (**c**) At follow-up CT performed 1 year later, there is a remarkable increase of subpleural opacity in the right lobe with the development of a 32-mm mass, resulting in lung adenocarcinoma (*arrow*)

The detection of lung cancer could also be affected by the location of the lesion when arising within previous lung abnormalities, such as interstitial lung disease. Sverzellati and colleagues [50] showed that important abnormalities consistent with fibrotic interstitial lung disease may be encountered in participants in a lung cancer screening trial, with the crude prevalence of usual interstitial pneumonia/other chronic interstitial pneumonia (UIP/OCIP) of 4.0 % (95 % CI 2.8–5.9%). In the NELSON Screening trial, signs of pulmonary fibrosis were reported in 117 (8 %) out of 1409 cases [51]. The presence of radiological signs of interstitial lung disease could delay the diagnosis of early stage lung cancer as tumors can be hidden or overlooked in the presence of underlying lung abnormalities (Fig. 12). This is even more important if we consider that idiopathic pulmonary fibrosis is associated with an increased risk of lung cancer [52] and some studies reported that the tumors preferentially occurred in areas with lung fibrosis [53, 54].

## Conclusion

There is a wide spectrum of low-dose CT findings in lung cancer screenings. Awareness of the various radiological features of early lung cancers and the familiarity with the low-dose CT technique can improve the CT screening effectiveness and avoid missed diagnosis.
